# Effects of *OsMSH6* Mutations on Microsatellite Stability and Homeologous Recombination in Rice

**DOI:** 10.3389/fpls.2020.00220

**Published:** 2020-03-03

**Authors:** Meng Jiang, Xiaojiang Wu, Yue Song, Hongzhe Shen, Hairui Cui

**Affiliations:** ^1^Key Laboratory of Chinese Ministry of Agriculture for Nuclear-Agricultural Sciences, Institute of Nuclear-Agricultural Science, Zhejiang University, Hangzhou, China; ^2^National Key Laboratory of Rice Biology, Institute of Crop Sciences, Zhejiang University, Hangzhou, China

**Keywords:** DNA mismatch repair, homeologous recombination, microsatellite stability, *OsMSH6*, rice (*Oryza sativa*)

## Abstract

DNA mismatch repair (MMR) system is important for maintaining DNA replication fidelity and genome stability by repairing erroneous deletions, insertions and mis-incorporation of bases. With the aim of deciphering the role of the MMR system in genome stability and recombination in rice, we investigated the function of *OsMSH6* gene, an import component of the MMR system. To achieve this goal, homeologous recombination and endogenous microsatellite stability were evaluated by using rice mutants carrying a *Tos17* insertion into the *OsMSH6* gene. Totally 60 microsatellites were analyzed and 15 distributed on chromosome 3, 6, 8, and 10 showed instability in three *OsMSH6* mutants, D6011, NF7784 and NF9010, compared with the wild type MSH6WT (the control). The disruption of *OsMSH6* gene is associated with modest increases in homeologous recombination, ranging from 2.0% to 32.5% on chromosome 1, 3, 9, and 10 in the BCF_2_ populations of the mutant ND6011 and NF9010. Our results suggest that the *OsMSH6* plays an important role in ensuring genome stability and genetic recombination, providing the first evidence for the *MSH6* gene in maintaining microsatellite stability and restricting homeologous recombination in plants.

## Introduction

Living cells respond to DNA damaging agents by modulating gene expression and arresting the cell cycle to ensure efficient DNA repair during their entire life. For maintaining the genetic stability and integrity, several DNA damage repair systems have been formed in the process of evolution (Schröpfer et al., 2014; Spampinato, 2017), one of which is mismatch repair (MMR). The MMR system is a major DNA repair pathway with ability to recognize and repair erroneous insertions, deletions and mis-incorporation of bases during DNA replication, genetic recombination and repair of some forms of DNA damage (Iyer et al., 2006; Jriecny, 2006; Spampanito et al., 2009). Plant MMR system consists of MutL and MutS mismatch repair homolog proteins, without MutH which is exclusively found in prokaryotes (Modrich, 1991; Fukui, 2010; Singh et al., 2010). As the most important monomers in MMR system, MSH2 and MLH1 form heterodimers with other MMR proteins, such as MutSα (MSH2-MSH6), MutSβ (MSH2-MSH3), MutSγ (MSH2-MSH7), MutLα (MLH1-PMS1), and MutLγ (MLH1-MLH3) (Ade et al., 1999; Culligan and Hays, 2000; Higgins et al., 2004; Li, 2008). MutSα is predominantly involved in the correction of short insertion/deletion loops (IDLs) and base-base mismatches, while MutSβ is preferentially required to remove the large IDLs (2-12 nucleotides), and plant specific MutSγ mainly recognizes single base mismatches (Culligan and Hays, 2000; Gomez and Spampinato, 2013).

Microsatellites, also called simple sequence repeats (SSRs), are simple tandem repeats of 1–6 bp DNA motifs and have been considered as a general source for polymorphic DNA markers widely used for multiple purposes in plants (Tautz, 1989; Varshney et al., 2005; Kalia et al., 2011). They are often subject to frequent unequal crossing over by misaligned pairing of repeats (Lovett, 2004) and inherently unstable as a consequence of a high level of DNA polymerase slippage on repetitive DNA during the replication process (Sia et al., 1997, 2001; Tran et al., 1997; Schlötterer, 2000). Such regions in genome are intrinsically unstable, causing size variation in the repeat sequences, contractions or expansions of the number of repeat units (Nag et al., 2004). As a type of molecular marker with several advantages, such as strong discriminatory power, co-dominant inheritance, and greater reproducibility (Varshney et al., 2005), SSR is suitable for monitoring genome instability. Microsatellite instability (MSI) is also considered as a consequence of MMR deficiency and has emerged as an important predictor of sensitivity for multiple human tumor types (Baretti and Le, 2018). As expected, studies have indicated that plant MMR deficiency also displayed MSI, such as deficiency of *MSH2* (Leonard et al., 2003; Hoffman et al., 2004) and *PMS1* (Alou et al., 2004; Xu et al., 2012).

Genetic recombination involves the genetic information exchange between highly similar DNA sequences, which can create both novel alleles and new combinations of alleles (Puchta and Hohn, 1996). Such novel variations can be useful both for plant adaptation to environment and variety improvement. Studies in yeast and mammalian have shown that the MMR system is involved in limiting recombination between diverged sequences by recognizing mismatches and interfering with the formation and/or extension of heteroduplex intermediates, triggering either helicase-driven unwinding or immediate resolution of the heteroduplex intermediates (Chen and Jinks-Robertson, 1999; Kolas and Cohen, 2004), a function that has been termed as anti-recombination (Surtees et al., 2004). The anti-recombination role of MMR genes are relatively well studied in the model plant *Arabidopsis thaliana*. The loss of AtMSH2 and AtPMS1 activities led to increases in homeologous recombination between sequences of varying divergence (Li et al., 2006, 2009). AtMSH2 was reported to be involved in the suppression of somatic recombination between divergent direct repeats and between homologs from different ecotypes (Emmanuel et al., 2006) and its mutation induced a significant increase in intrachromosomal recombination between highly diverged sequences in germinal tissues (Lafleuriel et al., 2007). Studies on anti-recombination function of MMR genes in other plants have been relatively limited, only reported in tomato and moss *Physcomitrella patens* (Trouiller et al., 2006; Tam et al., 2011).

Rice is an important food crop. Its MMR genes have been annotated in Rice Annotation Project Database (RAPD)^1^ and 12 MMR genes have been identified in rice genome using similarity searches and conserved domain analysis (Singh et al., 2010), one of which is *OsMSH6* (*LOC_Os09g24220*), a homolog to *AtMSH6* (*At4g02070*) in the MMR system of *A. thaliana*. However, no information is available for the *MSH6* gene function in maintaining genome stability in plants including *A. thaliana*. With the aim of deciphering its role in genetic stability and recombination, we have demonstrated that *OsMSH6* plays an important role in ensuring genome stability by recognizing mismatches arising spontaneously in rice (Cui et al., 2017). Herein, we reported the effects of *OsMSH6* disruption on microsatellite stability and homeologous recombination in rice.

## Materials and Methods

### Plant Materials

By a BLAST search against flanking sequences in Rice Tos17 Insertion Mutant Database^2^, insertion mutant seeds of *OsMSH6* (*LOC_Os09g24220*) gene were introduced from National Institute of Agrobiological Sciences, Japan. Three homozygous insertion mutants derived from Nipponbare, NF9010, NF7784 and ND6011 with the *Tos17* insertion position at 1st exon, 8th exon and 3′-UTR respectively, were obtained at T_3_ generation after molecular analyses (Li et al., 2012). The wild type, MSH6WT without *Tos17* insertion derived from the segregating generation, was used as the control to eliminate the mutations in mutants caused by the somaclonal variation during the tissue culture. Three BCF_2_ populations, obtained by crosses and backcrosses of MSH6WT, ND6011 or NF9010 with Zaoxian B (ZXB, an *indica* variety) respectively, were used for homeologous recombination assay.

### DNA Extraction and Mutant Identification

Genomic DNA was extracted from leaves according to a modified CTAB method described by Murray and Thompson (1980) and adjusted to a final concentration of 25 ng/μL after quantification using a Nanodrop 2000 spectrophotometry (Thermo Scientific, United States). The DNA quality was further confirmed by 1.5% agarose gel electrophoresis at 90 V for 30 min.

**FIGURE 1:**
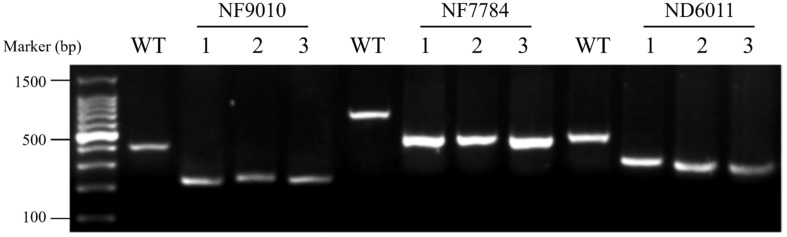
Agarose gel electrophoresis of triple-primer PCR products amplified from insertion mutants. WT: MSH6WT. 1 to 3: three independent plants from the insertion mutants of NF9010, NF7784, and ND6011, respectively. Product size: NF9010FP/NF9010RP (424 bp), TP/NF9010RP (249 bp), NF7784FP/NF7784RP (765 bp), TP/NF7784RP (524 bp), ND6011FP/ND6011RP (530 bp), TP/NF9010RP (342 bp).

The mutants were characterized by triple-primer PCR (polymerase chain reaction) (Figure 1). Reaction system (20 μL) contained ∼50 ng template DNA and 0.4 μM of each primer in 2 × Taq Master Mix (TOYOBO, Japan). The PCR program used included a hot start of 5 min at 94°C followed by 30 cycles of 45 s denaturation at 94°C, 45 s at 60°C (ND6011 for 58°C) annealing, 1 min polymerization at 72°C, and concluded by 10 min at 72°C. PCR products were detected by 1.5% agarose gel electrophoresis. Forward primer (FP) and reverse primer (RP) (Supplementary Table 1) at upstream and downstream of the *Tos17* insertion position were designed using the NCBI software^3^. *Tos17* insertion position primer (TP) (Supplementary Table 1), a specific sequence primer located at the conjunction of rice genome with the inserted *Tos17*, was adopted from the Gramene data resource^4^.

### RNA Extraction and Transcriptional Expression Assay

Total mRNA was extracted according to the method described by Jiang et al. (2019a; 2019b). Seedlings were cultured in 1 × Murashige and Skoog liquid medium (Murashige and Skoog, 1962) at 30°C under long day conditions (16 h/8 h light/dark). Total RNAs were extracted from leaf tissues of 10-day-old (10d), 15-day-old (15d) and 20-day-old (20d) seedlings at midday using the RNeasy Plant RNA Mini Kit (Qiagen, Germany). cDNAs were synthesized from 1 μg total RNA using the oligo(dT_18_) primer and GoScript^TM^ Reverse Transcription System Kit (Promega, United States).

**FIGURE 2:**
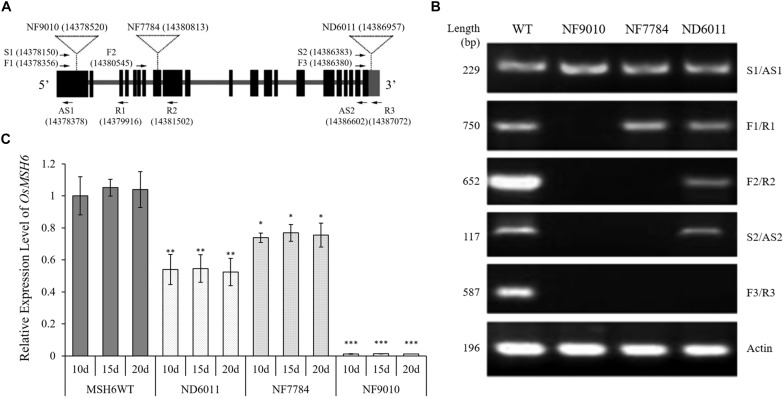
Identification of *Tos17* insertion mutants of *OsMSH6* gene. **(A)** Schematic diagram of *OsMSH6* with the positions of the inserted *Tos17* and primers (the number in brackets represents location of Tos17 insertion and primers on chromosome 9 in DNA Database IRGSP-1.0). **(B)** Reverse transcription analysis of *OsMSH6* in *OsMSH6* mutants (NF9010, NF7784 and ND6011) and MSH6WT. **(C)** Expression levels of *OsMSH6* in 10-day-old (10d), 15-day-old (15d), and 20-day-old (20d) were analyzed with six biological replicates. The expression level was first normalized to the internal control gene *OsACTIN* and reported relative to the expression level of *OsMSH6* in 10d MSHWT (assigned a value of 1). Error bars represent standard error. ^∗^, ^∗∗^, and ^∗∗∗^ represent significance between the materials and 10d MSHWT at *P* < 0.05, *P* < 0.01, and *P* < 0.001, respectively.

The sequences and the position of primers used for reverse transcription PCR (RT-PCR) were shown in Figure 2A and Supplementary Table 1. The RT-PCR system was performed using ∼25 ng template cDNA from 20-day-old plants, 0.5 μM of each primer, 10 μL 2 × Taq Master Mix (TOYOBO, Japan), and adding ddH2O to 20 μL. The PCR program used included a hot start of 5 min at 94°C followed by 35 cycles of 45 s denaturation at 94°C, 30 s at 55°C annealing, 1 min polymerization at 72°C, and concluded by 10 min at 72°C. The rice *ACTIN* gene (*OsACTIN, LOC_Os03g50885*) was used as control and the RT-PCR products were detected by 1.5% agarose gel electrophoresis.

The primer pair F1/R1 (Figure 2A and Supplementary Table 1) was used for quantitative real-time PCR (qRT-PCR) to detect the transcriptional expression level of *OsMSH6* by using a SYBR Green GoTaq^®^ qPCR Master Mix (Promega, United States). The *OsACTIN* gene was used as an internal control and relative expression levels were calculated using the 2^–ΔΔCt^ method (Livak and Schmittgen, 2001). All measurements were completed using six biological replicates, and statistical analyses were conducted using the Student’s *t*-test.

### Microsatellite Analysis

**FIGURE 3:**
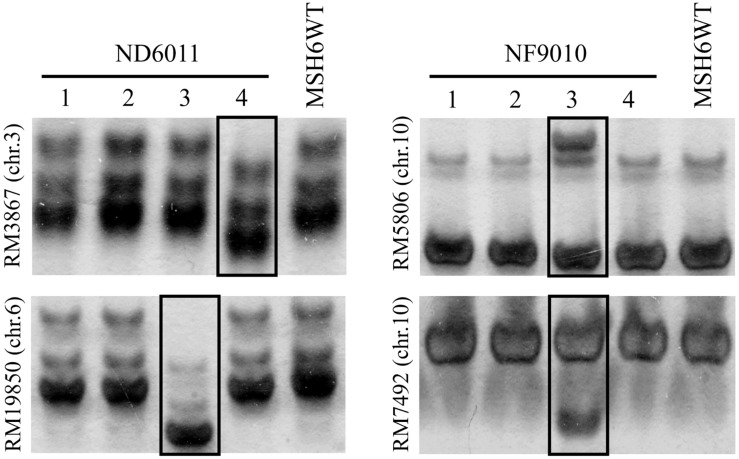
Examples of genomic stability in *OsMSH6* mutants and MSH6WT. 1 to 4: four independent lines. The varied microsatellite is marked in the box.

Four independent lines of each mutant in T_6_ generation were randomly selected for collection of leaf tissues. Leaf sample from eight plants in each line of mutants, as well as the control, were collected and mixed to extract DNA for SSR amplification by PCR. PCR reactions were carried out in a 15 μL volume containing ∼50 ng DNA and 0.4 μM of each primer in 2 × Taq Master Mix (TOYOBO, Japan). The PCR program used included a hot start of 5 min at 94°C followed by 30 cycles of 1 min denaturation at 94°C, 1 min at suitable temperature annealing, 1 min polymerization at 72°C, and concluded by 10 min at 72°C. Sixty microsatellites distributed on chromosomes 1, 3 6, 8, 9, and 10 were randomly selected and analyzed for stability. Sequences of the various primers used in present study were showed in Supplementary Table 2. The PCR products were then separated by 8% polyacrylamide gel electrophoresis (Figure 3) and visualized by silver-staining according to Liu et al. (2007).

### Homeologous Recombination Assay

**FIGURE 4:**
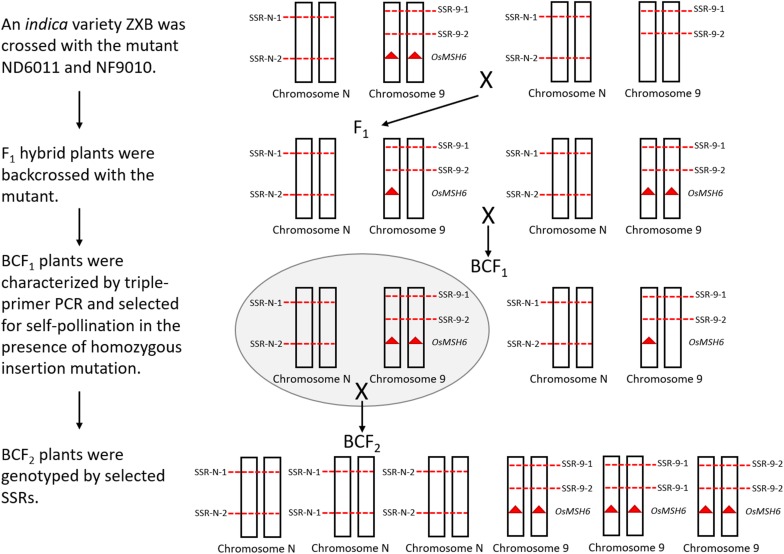
A whole schematic figure explaining how the segregating population was obtained. The mutants with *Tos 17* inserted *OsMSH6* gene mutation (red triangle) is on the left side in the top row. Chromosome 9 represents the *OsMSH6* gene located chromosome, and Chromosome N represents other chromosomes (1, 3, and 10). SSR-N represents an SSR marker located on Chromosome N, and SSR-9 represents an SSR marker located on Chromosome 9. On the right side in the top row is an *indica* variety ZXB without *Tos 17* inserted *OsMSH6* gene mutation.

Sixty-eight SSRs distributed on chromosome 1, 3, 9, and 10 were screened to detect polymorphism between ZXB and each mutant or the control. Ten polymorphic SSRs relatively evenly distributed on each chromosome mentioned above were selected to mapping for three BCF_2_ populations (Figure 4). An *indica* variety ZXB was crossed with the mutants ND6011 and NF9010. F_1_ hybrid plants were backcrossed with the mutants. BCF_1_ plants were characterized by triple-primer PCR and selected for self-pollination in the presence of homozygous insertion mutation. Meanwhile, a BCF_2_ segregating population was also constructed with the wild type (MSH6WT) background as the control. For each BCF_2_ population (ND6011/ZXB, NF9010/ZXB and MSH6WT/ZXB), 200 plants were genotyped using the polymorphic SSRs (Supplementary Table 3). MAPMAKER version 3.0 was used to estimate recombination fractions using a threshold LOD score of 3.0 for detecting linkage. Map distances were calculated using the Kosambi mapping function. Results from different populations (the wild type control and mutant populations) were compared for significant differences using the *t*-test.

## Results

### Molecular Identification of Homozygous Insertion Mutants

Three *Tos17* insertion mutants, NF9010, NF7784, and ND6011 were characterized by triple-primer PCR and RT-PCR. The *Tos17* insertions in *OsMSH6* gene were confirmed as homozygous state using triple-primer PCR method (Figure 1). In both NF9010 and NF7784 mutant seedlings, partial *OsMSH6* mRNA transcripts were detected with primer sets in upstream region of the insertion site (Figure 2B). However, it was not detectable with primer sets in the downstream region or across the insertion site, indicating that NF9010 and NF7784 lacked full length functional mRNA. Transcripts were detectable with primer sets in the upstream, internal and downstream fragments of coding region in ND6011 mutant, but no transcript was detected with the primer set across the insertion site, suggesting that mutant ND6011 lacked a fragment in 3′ untranslated region (Figure 2B).

We further detected *OsMSH6* expression levels in seedlings at different growth time by qRT-PCR (Figure 2C). The results revealed significant difference of *OsMSH6* expression levels between the *OsMSH6* mutant and MSH6WT, while no difference among different growth time (10d, 15d, and 20d) was observed in each mutant or control (Figure 2C).

### Microsatellite Instability in the *OsMSH6* Mutants

Sixty mapped SSR primer pairs (Supplementary Table 2) were used to investigate possible microsatellite instability in three mutant lines. Out of sixty microsatellites totally analyzed, 15 (25%) SSRs were polymorphic between *OsMSH6* mutant and MSH6WT (Table 1), with nine, six and two in NF9010, ND6011, and NF7784, respectively. The polymorphic forms included not only the increasing number of bands, but also the displacement of the main band position (Figure 3). The polymorphism suggested that SSR variations were induced by the *OsMSH6* mutation.

**TABLE 1:** The microsatellites displayed variations in *OsMSH6* mutants.

**Microsatellites**	**Chromosome**	**Repeat motif**	**Variation**	**Mutants**
RM2334	3	(AT)25	Contraction	ND6011, NF9010
RM15362	3	(TC)12	Contraction	ND6011
RM3867	3	(GA)30	Contraction	ND6011, NF9010
RM19850	6	(CT)15	Contraction	ND6011
RM8271	8	(AG)32	Contraction	NF9010
RM6027	8	(CCG)8	Contraction	NF9010
RM25701	10	(CT)13	Contraction	NF7784
RM7492	10	(TATC)7	Contraction	NF9010
RM25271	10	(TC)17	Contraction	NF9010
RM3291	3	(CT)14	Expansion	NF7784
RM6676	3	(TAA)8	Expansion	NF9010
RM3724	6	(GA)16	Expansion	ND6011
RM7311	6	(CAAT)6	Expansion	ND6011
RM5806	10	(AGG)9	Expansion	NF9010
RM271	10	(GA)15	Expansion	NF9010

We further analyzed the location, repeat motifs and direction of microsatellite variation (Table 1). No polymorphic microsatellite was found on chromosome 1 and 9, while five, three, two, and five polymorphic SSRs were found on chromosome 3, 6, 8, and 10, respectively. Theses varied microsatellites included 10 di-nucleotide-repeats, three tri-nucleotide repeats and two tetra-nucleotide-repeats, accounting for 26.3% (10/38), 16.7% (3/18), and 50.0% (2/4), respectively. Totally we analyzed 29 types of repeat motifs and found ten (34.5%) displayed variations in repeat length, including five di-nucleotide-repeat motifs (GA, CT, AG, TC, and AT), three tri-nucleotide-repeat motifs (CCG, TAA, and AGG) and two tetra-nucleotide-repeat motifs (TATC and CAAT). Among these fifteen varied microsatellites, nine (60%) showed contraction variations and six (40%) showed expansions. On average, the original length of repeats showing contraction and expansion was 37.8 and 27.5 bp, respectively.

### Effects on Homeologous Recombination

**FIGURE 5:**
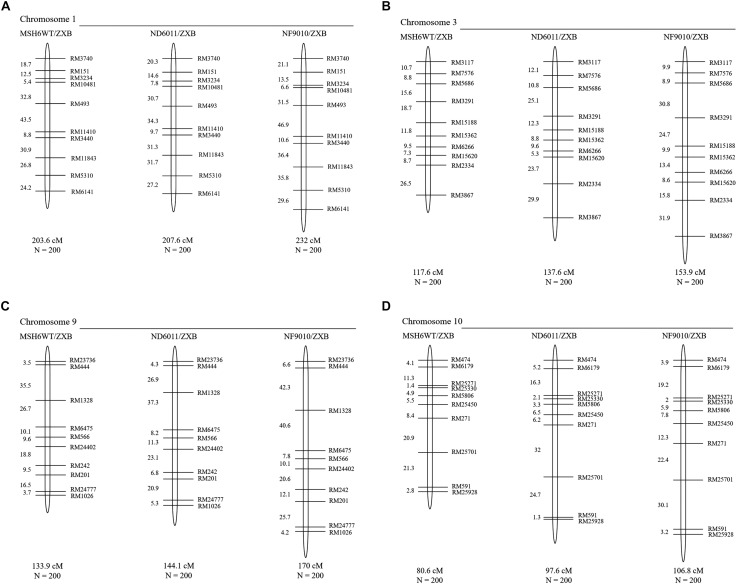
Genetic linkage maps of MSH6WT/ZXB, ND6011/ZXB and NF9010/ZXB based on recombination in BCF_2_ progeny. Genetic distances are in Kosambi map units, with total genetic length (cM) and the total number of individuals (N) in each BCF_2_ mapping population represented below the chromosome. **(A–D)** Represent the genetic linkage maps of chromosome 1, 3, 9 and 10, respectively.

The total map length of four chromosomes was about 535.7 cM in the control (MSH6MT/ZXB) population, with 203.6 cM, 117.6 cM, 133.9 cM and 80.6 cM for chromosome 1, 3, 9, and 10, respectively (Figure 5). When compared to the control population, homeologous recombination was found to be increased in two BCF_2_ populations with the mutant background (Figure 5 and Table 2). In ND6011/ZXB population, the total map length was increased by 51.2 cM (9.6%), but it was not significant. However, a significant increase (77.6 cM, 13.2%) was observed in total map length for NF9010/ZXB population. These results indicated that *OsMSH6* plays an important role in restricting homeologous recombination.

**TABLE 2:** Homeologous recombination in BCF_2_ populations derived from ZXB (a variety of *indica* rice) crossed and backcrossed with Msh6WT (control), ND6011, NF9010.

**Chromosome**	**Marker interval (cM)**	**MSH6WT/ZXB (control)**	**ND6011/ZXB**	**NF9010/ZXB**
1	RM3740 - RM151	18.7	20.3	21.1
	RM151 – RM3234	12.5	14.6	13.5
	RM3234 – RM10481	5.4	7.8	6.6
	RM10481 – RM493	32.8	30.7	31.5
	RM493 – RM11410	43.5	34.3	46.9
	RM11410 – RM3440	8.8	9.7	10.6
	RM3440 – RM11843	30.9	31.3	36.4
	RM11843 – RM5310	26.8	31.7	35.8
	RM5310 – RM6141	24.2	27.2	29.6
Total map length (cM)		203.6	207.6	232
% Change from control			1.96 (*P* = 0.376)	13.95** (*P* = 0.008)
3	RM3117 – RM7576	10.7	12.1	9.9
	RM7576 – RM5686	8.8	10.8	8.9
	RM5686 – RM3291	15.6	25.1	30.8
	RM3291 – RM15188	18.7	12.3	24.7
	RM15188 – RM15362	11.8	8.8	9.9
	RM15362 – RM6266	9.5	9.6	13.4
	RM6266 – RM15620	7.3	5.3	8.6
	RM15620 – RM2334	8.7	23.7	15.8
	RM2334 – RM3867	26.5	29.9	31.9
Total map length (cM)		117.6	137.6	153.9
% Change from control			17.01 (*P* = 0.169)	30.87* (*P* = 0.025)
9	RM23736 – RM444	3.5	4.3	6.6
	RM444 – RM1328	35.5	26.9	42.3
	RM1328 – RM6475	26.7	37.3	40.6
	RM6475 —- RM566	10.1	8.2	7.8
	RM566 – RM24402	9.6	11.3	10.1
	RM24402 – RM242	18.8	23.1	20.6
	RM242 – RM201	9.5	6.8	12.1
	RM201 – RM24777	16.5	20.9	25.7
	RM24777 – RM1026	3.7	5.3	4.2
Total map length (cM)		133.9	144.1	170
% Change from control			7.62 (*P* = 0.272)	26.96* (*P* = 0.022)
10	RM474 – RM6179	4.1	5.2	3.9
	RM6179 – RM25271	11.3	16.3	19.2
	RM25271 – RM25330	1.4	2.1	2
	RM25330 – RM5806	4.9	3.3	5.9
	RM5806 – RM25450	5.5	6.5	7.8
	RM25450 – RM271	8.4	6.2	12.3
	RM271 – RM25701	20.9	32	22.4
	RM25701 – RM591	21.3	24.7	30.1
	RM591 – RM25928	2.8	1.3	3.2
Total map length (cM)		80.6	97.6	106.8
% Change from control			21.09 (*P* = 0.106)	32.51* (*P* = 0.015)

Level of statistical significance *P < 0.05 and **P < 0.01. *P-*values given are for one-tailed test.

The increase rates varied with the chromosome. In ND6011/ZXB population, the map length of chromosome 1, 3, 9, and 10 was increased by 1.96% (4.0 cM, *P* = 0.376), 17.01% (20.0 cM, *P* = 0.169), 7.62% (10.2 cM, *P* = 0.272) and 21.09% (17.0 cM, *P* = 0.106), respectively, though not significant for all chromosomes. While in NF9010/ZXB population, the increase rate was 13.95% (28.4 cM, *P* = 0.008), 30.87% (36.3 cM, *P* = 0.025), 26.96% (36.1 cM, *P* = 0.022), and 32.51% (28.0 cM, *P* = 0.015) for chromosome 1, 3, 9, and 10, respectively (Table 2). All chromosomes showed a same tendency of increase rates in the two populations with the mutant background: the largest increase rate on chromosome 10 followed in order by chromosome 3, chromosome 9 and chromosome 1.

Compared to the control, different marker intervals did not show consistent changes in two BCF_2_ populations with the mutant background. Totally 36 marker intervals were assessed. Generally, the NF9010/ZXB BCF_2_ population showed more increased marker intervals (31) in map distances than the ND6011/ZXB BCF_2_ population (25). Twenty-six marker intervals were only consistently observed in the two populations, 23 showing increases and 3 showing decreases in map length. For the remaining 10 marker intervals, no particular trend was apparent with one population recording increases while another showing decreases in map distances (Figure 5 and Table 2).

## Discussion

As MSH6, an important part of the MMR system, can repair a variety of deletions, insertions and mis-incorporation created during DNA synthesis (Iyer et al., 2006; Li, 2008; Spampanito et al., 2009), it would be expected that the inactivation of *OsMSH6* could impact the genome stability and increased recombination rates in rice. Indeed, we observed the MSI in the genome and increased recombination rates on four chromosomes under OsMSH6 mutation background.

DNA mismatch repair (MMR) deficiency can cause dissociation of the nascent strand and the template during replication, and high mutation rate in microsatellites by failing to correct replication errors (Schlötterer, 2000; Li et al., 2002; Hawk et al., 2005). It has been reported that microsatellite instability can be used as a hallmark of MMR gene deficiency (Sia et al., 1997). DNA mismatch repair (MMR) deficiency results in a strong mutator phenotype and high-frequency microsatellite instability (MSI-H) in *Homo sapiens* and other species (Imai and Yamamoto, 2008). In plants, MSI has often been related to MMR deficiency. For example, deficiency of *MSH2* and *PMS1* may cause high MSI (Leonard et al., 2003; Alou et al., 2004; Hoffman et al., 2004; Xu et al., 2012). Our result is similar to these reports mentioned above. We examined 60 microsatellites and found fifteen polymorphic SSRs between *OsMSH6* mutants and MSH6WT (Table 1), which revealed that *OsMSH6* mutations induced microsatellite instability. Thus, it demonstrated that the *OsMSH6* is an MMR gene that plays an important role in maintaining SSR stability. The result also further proves the conclusion in our previous research that the *OsMSH6* is important in ensuring genome stability by recognizing and repairing mismatches that arise spontaneously in rice by specific-locus amplified fragment (SLAF) sequencing (Cui et al., 2017). Among the polymorphic microsatellites observed in this research, the number of contractions SSR was a little higher than the expansion (Table 1), but the difference was not significant. So, it seemed no obvious preference to contraction or expansion of SSR variations induced by *OsMSH6* mutation in rice.

In present research, we found that disruption of rice MMR gene *OsMSH6* by *Tos 17* insertion was associated with modest but significant increase rate in homeologous recombination (Figure 5 and Table 2). This result was consistent with the effects of other MMR genes reported in different plants, such as *MSH2* in *Physcomitrella patens* (Trouiller et al., 2006), *Arabidopsis* (Emmanuel et al., 2006; Li et al., 2006, 2009; Lafleuriel et al., 2007) and tomato (Tam et al., 2011), *MSH7* in tomato (Tam et al., 2011), *MLH1* and *PMS1* in *Arabidopsis* (Dion et al., 2007; Li et al., 2009). It was proved that the MMR gene *MSH6* has an important anti-recombination role in yeast (Nicholson et al., 2000) and mice (Barreraoro et al., 2008). However, its possible role in homeologous recombination has not been investigated in plants. Here our data in manner of map distance showed the anti-recombination activity of *MSH6* gene in rice. Disruption of *OsMSH6* resulted in significantly increased recombination rates (9.6 and 13.2% greater than controls for two mutants) (Table 2). Modest increases in recombination rates observed in our study suggest that the *OsMSH6* is a minor suppressor of homeologous recombination.

The increased rate in homeologous recombination observed in our study, ranging from 2.0 to 32.5% for different chromosomes compared to the control (Table 2), was similar to that reported in tomato (Tam et al., 2011). However, it was much lower than reported in *Arabidopsis thaliana* (Dion et al., 2007; Lafleuriel et al., 2007; Li et al., 2006, 2009). Different magnitudes of increase in homeologous recombination in our research could be caused by several factors.

One is that we investigated a different MMR gene. The MSH2 is a key subunit in MMR and the constant component of the heterodimers formed with MutS proteins such as MSH3, MSH6, and MSH7 (Harfe and Jinks-Robertson, 2000; Wu et al., 2003). Therefore, the MSH2 is essential for all kinds of mismatch recognition complexes to function and its deficiency would be expected to cause bigger effects on recombination rates compared to MSH3, MSH6, or MSH7. Indeed, it has been reported that MSH2 showed higher anti-recombination activity than MSH3, MSH6, and MLH1 in yeast (Chambers et al., 1996; Nicholson et al., 2000) or PMS1 (Selva et al., 1995; Datta et al., 1996) and bigger effects were also observed on recombination rates for MSH2 inactivation, resulting in recombination rate increases by 3-fold (Lafleuriel et al., 2007) or 2 to 7-fold (Li et al., 2006) in *A. thaliana*. In this study we found its disruption caused recombination rate increase by 2.0 to 32.5% for different chromosomes in rice, which is much lower than that by *MSH2* inactivation as mentioned above in *A. thaliana*. The differences in suppression or expression level of MMR genes may also cause different magnitudes of increase in recombination rates. For example, reduced *MSH2* gene expression in tomato via RNAi-induced silencing or dominant negative regulating only showed a modest increase, ranging from 3.8 to 29.2%, lower than that caused by *MSH2* inactivation in *A. thaliana* (Tam et al., 2011). We used two mutants NF9010 and ND6011 for homeologous recombination essay. No functional *OsMSH6* mRNA was detected (Figure 2) and significant increases in recombination rates (Figure 5) were observed for the mutant NF9010, while reduced *OsMSH6* expression level and less increases in recombination rate were detected for the mutant ND6011. It also suggests that magnitudes of increase in homeologous recombination are related with the disruption or decreasing expression level of MMR gene.

Another important factor is the DNA divergence degree that may cause different magnitudes of recombination increase. Since DNA homology guides meiotic chromosome pairing, the efficiency of homologous can be dramatically reduced by sequence divergence between chromosomes in plants (Bozza and Pawlowski, 2008). MMR proteins inhibit recombination between highly diverged DNA sequences, which requires a less stringent requirement for DNA strand complementarity and higher tolerance for mismatches when recombination intermediates form during recombinational strand exchange/strand transfer (Worth et al., 1994). Trouiller et al. (2006) reported that 3% divergence between recombination substrates leads to a reduction of about 20% in the frequency of gene targeting in wild type moss while no reduction was observed in an *MSH2* mutant. Li et al. (2006) showed that the loss of AtMSH2 activity leads to a 2 to 9-fold increase in the frequency of intrachromosomal recombination with constructs containing 0.5, 2, 4, or 9% divergence between the recombination substrates. Lafleuriel et al. (2007) reported *MSH2* mutation caused a three-fold increase in intrachromosomal recombination between highly diverged sequences (13%) in germinal tissues. Based on the reports mentioned above, the increase in recombination frequency by *AtMSH2* mutation was smaller at low levels of divergence, but it became higher with more linear at higher levels of DNA divergence in *A. thaliana*. We checked the average SNP number per Kb on chromosome 1, 3, 9, and 10 between *Nippobare* (a *japonica* variety) and *Kalasath* (an *indica* variety) using SNPseek^5^. The SNP density on chromosome 1, 3, 9, and 10 was about 10.7, 11.3, 11.7, and 12.5/Kb, respectively. Compared with the sequence divergences listed in reports mentioned above, the divergences were lower as displayed in SNP density on four chromosomes involved in our research. Furthermore, we observed the smallest increase in chromosome 1 (1.96 and 13.95) and the largest increase in chromosome 10 (21.09 and 32.51) in two populations with the mutant background, which fit in well with the divergences of the two chromosomes. Thus, smaller increase rates in recombination could be caused by the lower degree of sequence divergences.

Understanding of recombination mechanisms would greatly facilitate variety improvement by transfers of genetic material from related species (Li et al., 2007; Martinez-Perez and Moore, 2008; Qi et al., 2007; Wijnker and de Jong, 2008). Because of depleted genetic diversity in many crop plants, wide crossing has been considered as an important strategy for crop genetic improvement (Tanksley and McCouch, 1997). In Asian cultivated rice (*Oryza sativa* L.), genetic differentiation between *indica* and *japonica* subspecies appears to be a major source of genetic diversity. The intersubspecific *indica-japonica* hybridization has been proposed as an important strategy for rice breeding and *indica-japonica* hybrids demonstrate strong heterosis with great promise for breeding of super rice (Lin et al., 2016; Ouyang, 2016). However, the *indica-japonica* hybridization is hindered by reproductive isolation (Ouyang and Zhang, 2013). Our results suggest that the *OsMSH6* mutation can increase homeologous recombination, thus to promote exchanges of genetic material between *indica* and *japonica* subspecies, as well as the introgression of beneficial genes from distantly related species. Therefore, the *OsMSH6* mutant is significant to such rice breeding programs.

## Data Availability Statement

All datasets generated for this study are included in the article/Supplementary Material.

## Author Contributions

HC designed the research work. MJ, YS, XW, and HS performed the experiments. MJ and YS analyzed the data together with HC. MJ drafted the manuscript. HC improved the manuscript.

## Conflict of Interest

The authors declare that the research was conducted in the absence of any commercial or financial relationships that could be construed as a potential conflict of interest.

## Footnotes

1 http://rapdb.dna.affrc.go.jp

2 http://tos.nias.affrc.go.jp

3 https://www.ncbi.nlm.nih.gov/tools/primer-blast

4 http://www.gramene.org

5 http://snp-seek.irri.org/_snp.zul
