# Supplementary material for: Effects of OsMSH6 Mutations on Microsatellite Stability and Homeologous Recombination in Rice
Source: Front Plant Sci. 2020 Mar 3;11:220. doi: 10.3389/fpls.2020.00220 (PMC7062918; doi:10.3389/fpls.2020.00220)
Supplement: Supplementary file 1 [file Data_Sheet_1.PDF]

Supplementary Table 1. Primers for triple-primer PCR identification and RT-PCR analysis of *Tos17* insertion mutant lines.

**Prime code**	**Primer sequence (5'-3')**
1. Primers for triple-primer PCR identification	
NF9010-FP	GTACTGGCCGCTGGACGACG
NF9010-RP	CGACGTCGACAAAGGGTTAT
NF7784-FP	TCCGCCGTAAAGAAACTGGT
NF7784-RP	CACCAGGTGTGTCCAATGAG
ND6011-FP	GCAACCATCTACCAAGCATT
ND6011-RP	GCGACCCGGTATAACAAAGA
TP	ATTGTTAGGTTGCAAGTTAGTTAAGA
2. Primers for RT-PCR analysis	
S1	TCCGCAAGCAGAGCCAGAT
AS1	ACGTCGTAGCCCTCCACCC
F1	GGGAGGGTGGAGGGCTACGA
R1	CAAGAACTGGGAAGGCAAGG
F2	CTTGACCTCCGCCGTAAAGA
R2	GGTTGGTGAGCCCTGAGCAT
S2	AGAAGGAGGTTTGGAAGAAG
AS2	TGAAGCACTGATGCAGGTAT
F3	GGGAGAAGGAGGTTTGGAAG
R3	AGAGTGCGGAGAAATGTTGC
Actin FP	CTTCATAGGAATGGAAGCTGCGGGTA
Actin RP	CGACCACCTTGATCTTCATGCTGCTA

Supplementary Table 2. Primers for microsatellite stability assay.

Chromosome	Microsatellite	Repeat motif	Primer forward	Primer reverse
1	RM3740	(GA)16	ATCCCAACTCTAAGCCACCC	CTACCCGTCACCAACTCACC
1	RM151	(TA)23	TGCTGATCAGTTACACGAATCAGAGC	GCGTACGTGCACAAATTAAACAGACC
1	RM3234	(CT)13	AAAGACGACGATGGGTCAAC	GTGAGGTTCTTGGGTGGAAG
1	RM10481	(AT)11	TGGAGAGAAGACAGAGCATATACTGG	CAGGGATGGAGCATTGGTAGG
1	RM493	(CTT)9	TAGCTCCAACAGGATCGACC	GTACGTAAACGCGGAAGGTG
1	RM11410	(GA)10	CATAACCCTGAAGTGGTGTGACG	GTGCTTGATGATATGGTCCTTGC
1	RM3440	(CT)19	TGTTCGAAGGAAGCTGGATC	TAGCTGAAGCCAAAGCCTTC
1	RM11843	(AGC)7	ACGCAGAATTGATTCGCTCTCG	AGAGGAAGAGGAGGGAACGAACG
1	RM5310	(TC)12	TAGACAAAGCAACGGGTTCC	CGGAAGCAGGAGAATCGTAG
1	RM6141	(CGC)9	AAGCTTCCCCAATCTGGAAC	TAGCTTAGCTGCTGCTGCTG
3	RM3117	(CA)12	GCCATCTCTCTCTCTCTCTCTC	CCTTAGCTCATCAAGCGAGG
3	RM7576	(TCTA)8	CTGCCCTGCCTTTTGTACAC	GCGAGCATTCTTTCTTCCAC
3	RM5686	(AAT)17	CTCTTCTATGCATATTGCCA	ATAAACTGAGGGGCGATATA
3	RM3291	(CT)14	CCTGCACAACAACAACAACC	TGTCTGTCCATCGATCCATG
3	RM6676	(TAA)8	AATGTTCACGGTCCAATAAG	CATGCATAACACCCAAATG
3	RM15188	(TTA)10	AGCCTCCTAGAACGGCAAAGG	CTATGTTTCGTGCAACCAAGTCC
3	RM15362	(TC)12	CTCGACACAAAGATCAGCAATGG	CTCAACCTCATCATCGCTTGC
3	RM15620	(AT)15	TTTGTTTAGCAGGAGGACCATGC	AGCCGGTGATCACGATTTGG
3	RM2334	(AT)25	CATGCATCTGATCTGATTAT	TGTGAAGAGTACAAGTAGGG
3	RM3867	(GA)30	TTGACTGGAACATCGAGCTC	ATCCCCTCTACACCGTACCC
6	RM276	(GA)33	CTCAACGTTGACACCTCGTG	TCCTCCATCGAGCAGTATCA
6	RM3724	(GA)16	TTCCCCGATTTTAGCTAGGC	GCGCAGAACACTGGCAAC
6	RM19795	(ATA)14	TAGTAGTTGGCATCTCCGGTTGC	CAAGCGGCCACTACGTATAGTACC
6	RM527	(GA)17	GGCTCGATCTAGAAAATCCG	TTGCACAGGTTGCGATAGAG
6	RM19815	(AAC)25	AAACGGAGGGAGTACATTG	GGATCGTCGATTTGATTTG
6	RM7213	(ATAG)9	CTATAGCCAGCGACGAGGAC	CTGCACCCATCTCTCTCTCC

6	RM7311	(CAAT)6	AGTGGTCGTTGAACTCGGAG	TCGTGGCGCCTTTAATCTC
6	RM19850	(CT)15	GATGTTCTCTCGGTTTGGACTTCG	CGACGAACAACAACTCAACTTTCACC
6	RM19955	(AT)21	CACCGCACGTGTATTGTTATATCC	TTTCTACCGCCAACCATTGC
6	RM19958	(AT)42	AACTAAAGGAGGTGGGCATTGG	GATACGTCATCCGTATTTGAGTCG
8	RM22581	(AT)10	GATACAAAGCATGTCTCCATCC	GGATGTTGGGTCTAAGAGTAGGG
8	RM22586	(CCG)7	TCACAAGGCCCACTTAATCTTCG	GCTGCTCGGTGAGGATGACC
8	RM3507	(CT)26	ACCCCTATCGATCAACCCTC	TTCGTTTGGTGTTAGGGGC
8	RM3644	(GA)14	GAAGAGAGTGGGAGGATGGG	AATTTGTGTGCTCCTCCACC
8	RM8271	(AG)32	TCTTGAGAAATCTGCCATTC	ACTGATGTGCATTTCGTC
8	RM22664	(CTG)7	CACTGCTGCCAACATCCATTCC	AAGGAAGAAGTGAAGTGGCATACGG
8	RM22685	(GGA)10	ATGGGCTTCCAGGCTCAATCTCG	CCCACTCTCACGTCTCCTCTCTTCC
8	RM8243	(CA)11	CTCGTGCAACCATTATATTC	ACCTTAGCTGTCCTGAATTG
8	RM6027	(CCG)8	AAGCTCAACAGCTACCTCGG	CCCGTACACCACCGGAAAC
8	RM3481	(CT)22	CTCGTCGCGTTCGTCAAC	CATCTCATCACCTCACGTCG
9	RM23736	(AT)44	GGCGATACCTGCCATAGTTTCC	CCGAAAGCAATCTATGAGACACC
9	RM444	(AT)12	GCTCCACCTGCTTAAGCATC	TGAAGACCATGTTCTGCAGG
9	RM1328	(AG)20	GAATGGGATTAGACGATTTG	CCATGAGTGACATCAAAAGG
9	RM6475	(GCC)9	AGATCAAAGCAACGGCTAGC	GAACAGAGAGGGGACGTGTC
9	RM566	(AG)15	ACCCAACTACGATCAGCTCG	CTCCAGGAACACGCTCTTTC
9	RM24402	(GGT)7	GTGCCACCACGACACCTTTGG	TAGAGGCCGTGGTTCCAGTGACG
9	RM242	(CT)26	GGCCAACGTGTGTATGTCTC	TATATGCCAAGACGGATGGG
9	RM201	(CT)17	CTCGTTTATTACCTACAGTACC	CTACCTCCTTTCTAGACCGATA
9	RM24777	(TTC)7	GTGGTGGTGGTGGACATCATGG	CAACGAGCTTTGTGAGACTGTGAGC
9	RM1026	(AC)14	GCCTCTGGCAGAATAGCATC	TATCACTTTGCTGCCTAGGC
10	RM7492	(TATC)7	AGATGGTTGCCAAGAGCATG	GTCACGTGGCGATTTAGGAG
10	RM6179	(CGG)8	ATCTCGTCCATCTCCGGC	TCCAACGGTCAAGATTAGCC
10	RM25271	(TC)17	AGACGCTACTCCCACCTGTAACC	ATATCATTGCCGCAACACAAGC
10	RM25330	(AG)17	ATCCAGCCACTGGTAGTCACAATACG	ATGAGGAGGCGCAGGATCACC
10	RM5806	(AGG)9	CTAATTGCGGTTGAAGCCTC	CCTCCCAATCTTTGCACATC

10	RM25450	(TC)18	CTTATCTCAAGCCCAATCACAACC	GATCTAGCTCCGCCACTCTCG
10	RM271	(GA)15	TCAGATCTACAATTCCATCC	TCGGTGAGACCTAGAGAGCC
10	RM25701	(CT)13	CGGCCTTATCCCTATATGAGTCAACC	CACGCGCGATAAGTGAGAGAGC
10	RM591	(AC)10	CTAGCTAGCTGGCACCAGTG	TGGAGTCCGTGTTGTAGTCG
10	RM25928	(GAA)7	AGGCAGCTAAGCTAGGCTAAAGG	GTCGATCTCACACTTCCGTTCC

Supplementary Table 3. Primers for homeologous recombination assay.

Chromosome	Marker name	Repeat motif	Primer forward	Primer reverse
1	RM3740	(GA)16	ATCCCAACTCTAAGCCACCC	CTACCCGTCACCAACTCACC
1	RM151	(TA)23	TGCTGATCAGTTACACGAATCAGAGC	GCGTACGTGCACAAATTAAACAGACC
1	RM3234	(CT)13	AAAGACGACGATGGGTCAAC	GTGAGGTTCTTGGGTGGAAG
1	RM10481	(AT)11	TGGAGAGAAGACAGAGCATATACTGG	CAGGGATGGAGCATTGGTAGG
1	RM493	(CTT)9	TAGCTCCAACAGGATCGACC	GTACGTAAACGCGGAAGGTG
1	RM11410	(GA)10	CATAACCCTGAAGTGGTGTGACG	GTGCTTGATGATATGGTCCTTGC
1	RM3440	(CT)19	TGTTCGAAGGAAGCTGGATC	TAGCTGAAGCCAAAGCCTTC
1	RM11843	(AGC)7	ACGCAGAATTGATTCGCTCTCG	AGAGGAAGAGGAGGGAACGAACG
1	RM5310	(TC)12	TAGACAAAGCAACGGGTTCC	CGGAAGCAGGAGAATCGTAG
1	RM6141	(CGC)9	AAGCTTCCCCAATCTGGAAC	TAGCTTAGCTGCTGCTGCTG
3	RM3117	(CA)12	GCCATCTCTCTCTCTCTCTCTC	CCTTAGCTCATCAAGCGAGG
3	RM7576	(TCTA)8	CTGCCCTGCCTTTTGTACAC	GCGAGCATTCTTTCTTCCAC
3	RM5686	(AAT)17	CTCTTCTATGCATATTGCCA	ATAAACTGAGGGGCGATATA
3	RM3291	(CT)14	CCTGCACAACAACAACAACC	TGTCTGTCCATCGATCCATG
3	RM15188	(TTA)10	AGCCTCCTAGAACGGCAAAGG	CTATGTTTCGTGCAACCAAGTCC
3	RM15362	(TC)12	CTCGACACAAAGATCAGCAATGG	CTCAACCTCATCATCGCTTGC
3	RM6266	(CTC)9	CCGTCACCTTCTTGAGAAGC	GACATCGAGAGCGAGGACAG
3	RM15620	(AT)15	TTTGTTTAGCAGGAGGACCATGC	AGCCGGTGATCACGATTTGG
3	RM2334	(AT)25	CATGCATCTGATCTGATTAT	TGTGAAGAGTACAAGTAGGG
3	RM3867	(GA)30	TTGACTGGAACATCGAGCTC	ATCCCCTCTACACCGTACCC
9	RM23736	(AT)44	GGCGATACCTGCCATAGTTTCC	CCGAAAGCAATCTATGAGACACC
9	RM444	(AT)12	GCTCCACCTGCTTAAGCATC	TGAAGACCATGTTCTGCAGG
9	RM1328	(AG)20	GAATGGGATTAGACGATTTG	CCATGAGTGACATCAAAAGG
9	RM6475	(GCC)9	AGATCAAAGCAACGGCTAGC	GAACAGAGAGGGGACGTGTC
9	RM566	(AG)15	ACCCAACTACGATCAGCTCG	CTCCAGGAACACGCTCTTTC
9	RM24402	(GGT)7	GTGCCACCACGACACCTTTGG	TAGAGGCCGTGGTTCCAGTGACG
9	RM242	(CT)26	GGCCAACGTGTGTATGTCTC	TATATGCCAAGACGGATGGG
9	RM201	(CT)17	CTCGTTTATTACCTACAGTACC	CTACCTCCTTTCTAGACCGATA
9	RM24777	(TTC)7	GTGGTGGTGGTGGACATCATGG	CAACGAGCTTTGTGAGACTGTGAGC
9	RM1026	(AC)14	GCCTCTGGCAGAATAGCATC	TATCACTTTGCTGCCTAGGC
10	RM474	(AT)13	AAGATGTACGGGTGGCATTC	TATGAGCTGGTGAGCAATGG
10	RM6179	(CGG)8	ATCTCGTCCATCTCCGGC	TCCAACGGTCAAGATTAGCC
10	RM25271	(TC)17	AGACGCTACTCCCACCTGTAACC	ATATCATTGCCGCAACACAAGC
10	RM25330	(AG)17	ATCCAGCCACTGGTAGTCACAATACG	ATGAGGAGGCGCAGGATCACC
10	RM5806	(AGG)9	CTAATTGCGGTTGAAGCCTC	CCTCCCAATCTTTGCACATC
10	RM25450	(TC)18	CTTATCTCAAGCCCAATCACAACC	GATCTAGCTCCGCCACTCTCG
10	RM271	(GA)15	TCAGATCTACAATTCCATCC	TCGGTGAGACCTAGAGAGCC
10	RM25701	(CT)13	CGGCCTTATCCCTATATGAGTCAACC	CACGCGCGATAAGTGAGAGAGC
10	RM591	(AC)10	CTAGCTAGCTGGCACCAGTG	TGGAGTCCGTGTTGTAGTCG
10	RM25928	(GAA)7	AGGCAGCTAAGCTAGGCTAAAGG	GTCGATCTCACACTTCCGTTCC
